# Diverse plasmid systems and their ecology across human gut metagenomes revealed by PlasX and MobMess

**DOI:** 10.1038/s41564-024-01610-3

**Published:** 2024-03-04

**Authors:** Michael K. Yu, Emily C. Fogarty, A. Murat Eren

**Affiliations:** 1 Toyota Technological Institute at Chicago, Chicago, IL USA; 2 Department of Medicine, University of Chicago, Chicago, IL USA; 3 Committee On Microbiology, University of Chicago, Chicago, IL USA; 4 Josephine Bay Paul Center for Comparative Molecular Biology and Evolution, Marine Biological Laboratory, Woods Hole, MA USA; 5 Alfred Wegener Institute for Polar and Marine Research, Bremerhaven, Germany; 6 Institute for Chemistry and Biology of the Marine Environment, University of Oldenburg, Oldenburg, Germany; 7 Helmholtz Institute for Functional Marine Biodiversity, Oldenburg, Germany; 8 Marine ‘Omics Group, Max Planck Institute for Marine Microbiology, Bremen, Germany

**Keywords:** Microbiology, Machine learning

## Abstract

Plasmids alter microbial evolution and lifestyles by mobilizing genes that often confer fitness in changing environments across clades. Yet our ecological and evolutionary understanding of naturally occurring plasmids is far from complete. Here we developed a machine-learning model, PlasX, which identified 68,350 non-redundant plasmids across human gut metagenomes and organized them into 1,169 evolutionarily cohesive ‘plasmid systems’ using our sequence containment-aware network-partitioning algorithm, MobMess. Individual plasmids were often country specific, yet most plasmid systems spanned across geographically distinct human populations. Cargo genes in plasmid systems included well-known determinants of fitness, such as antibiotic resistance, but also many others including enzymes involved in the biosynthesis of essential nutrients and modification of transfer RNAs, revealing a wide repertoire of likely fitness determinants in complex environments. Our study introduces computational tools to recognize and organize plasmids, and uncovers the ecological and evolutionary patterns of diverse plasmids in naturally occurring habitats through plasmid systems.

## Abstract (web summary)

A set of computational tools to de novo recognize plasmids in complex environments, like the human gut microbiome, and to organize them into evolutionarily cohesive units.

## Main

As a class of mobile genetic elements^1^, plasmids can occur in cells from all domains of life^2^, typically as extrachromosomal and circular DNA. Plasmids replicate semi-independently of their hosts and often transfer between cells as a mechanism of horizontal gene transfer^3^. A hallmark of plasmids is their remarkably diverse capacity to impact their microbial hosts through fitness-determining functions they carry^1^, such as antibiotic-resistance genes^4^ and virulence factors^5^. Plasmids also exhibit many interesting genetic properties, such as frequent recombination, which can result in plasmids sharing recurrent ‘backbone’ sequences but differing in their cargo genes^6–8^. These backbone sequences often encode for core replication and transfer machinery^6,7,9,10^ that determine the set of compatible hosts they can inhabit^10,11^ as well as regulate their copy number in a specific host^12^. Experiments in model systems and organisms in culture have revealed the critical impact of plasmids in microbial phenotypes and survival, especially for pathogens with medical significance. However, our understanding of the diversity, ecology and genetic architecture of naturally occurring plasmids are far from complete.

Recent advances in metagenomics offer unprecedented access to the entire DNA content of an environment without the need for cultivation. In particular, metagenomic assembly and binning strategies have enabled the reconstruction and characterization of microbial genomes de novo^13^, including those in the human gut where microorganisms have been associated with health and disease states^14^. Metagenomic approaches have also been applied to study plasmid content^15^, but such applications have been limited to shotgun sequencing of plasmid-enriched samples^16–18^ or to surveying only a handful of metagenomes at a time^19–21^. Over the past decade the number of publicly available metagenomes has rapidly increased, creating an opportunity to conduct large-scale studies to characterize the diversity of naturally occurring plasmids in complex ecosystems.

Several computational strategies have been developed to identify plasmids in sequence collections. Yet distinguishing plasmids from bacterial chromosomes or from other mobile genetic elements, such as viruses via computational strategies, remains a challenge^22^. Popular plasmid prediction strategies rely on *k*-mer patterns learned from reference plasmid sequences^19,20,23^, exploit known functions such as replication or conjugation genes^24–26^ or use a combination of these features^27^. Although these features can help identify plasmids similar to those in public databases, they are of limited utility to recognize plasmids that are not yet described. Other approaches focus on the circularity of sequences during (meta)genomic assembly^21,28,29^; however, this strategy overlooks plasmids that are linear, integrated or found as assembly fragments and may confuse other types of circular mobile elements for plasmids.

Here we present PlasX, a machine-learning approach to identify plasmids in complex microbial ecosystems, and Mobile Element Systems (MobMess), a robust network-partitioning algorithm to gain insights into plasmid evolution at scale. Using PlasX we identified a collection of 68,350 non-redundant plasmids in the human gut microbiome that were more genetically diverse than reference plasmids and substantially more prevalent across global human populations. We then used MobMess to organize predicted plasmids into ‘plasmid systems’ based on shared backbone sequences, which provided us with an evolutionary framework to investigate plasmid cargo gene content as a function of environmental pressures.

## Results

### Classification of plasmids based on de novo gene families

**Fig. 1:**
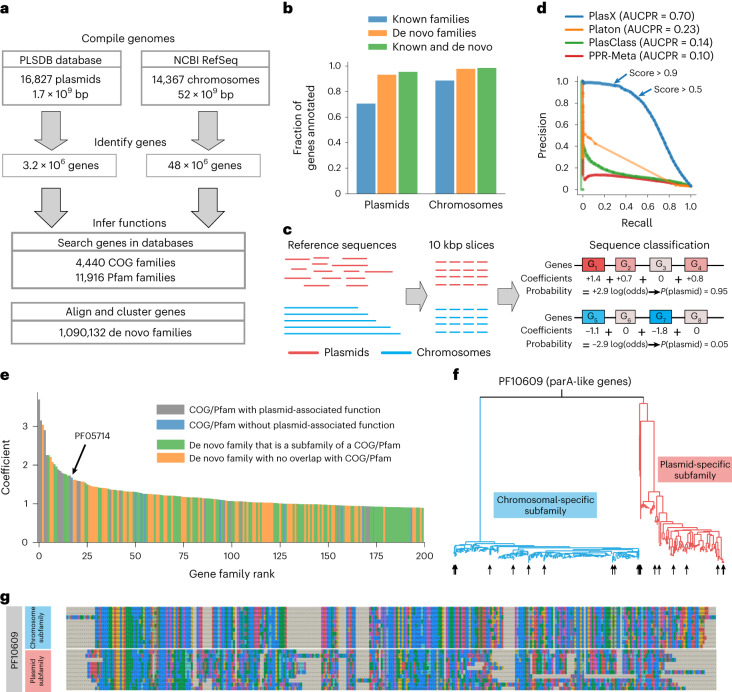
A machine-learning model for classifying plasmids. **a**, Our pangenomics workflow to characterize gene functions in a reference set of plasmids and chromosomes. **b**, Fraction of all plasmids or all chromosomal genes that are annotated using known families, de novo families or a combination of both. **c**, Training of PlasX. Reference sequences are sliced into 10-kbp windows and then prediction scores are made by a logistic regression that sums the contributions of gene families within a sequence. **d**, Precision–recall curves comparing PlasX, Platon, PlasClass and PPR-Meta. Except for PPR-Meta, every method was trained and evaluated using fourfold cross-validation and an informed split. The AUCPR value was calculated using sequence weights for normalization. The arrows indicate the performance of PlasX using a score threshold of either >0.5 or >0.9. **e**, Coefficients of the 200 gene families with the highest PlasX coefficients and that are thus most important for identifying plasmids. **f**, Maximum-likelihood phylogenetic tree of genes that are in PF10609 as well as either the plasmid-specific de novo subfamily mmseqs_5_1535552 or chromosome-specific de novo subfamily mmseqs_70_40217271. **g**, Sequence alignment of ten representative genes from each subfamily (arrows in **f**).

To train our machine-learning model, we first compiled a reference set of 16,827 plasmids and 14,367 chromosomal sequences from public databases (Fig. 1a and Supplementary Table 1), in which we identified 51.2 million open reading frames. We were able to assign a function to 71% of plasmid genes using the Clusters of Orthologous Groups (COG)^30^ and/or Pfam^31^ databases. In parallel, we clustered all genes into 1,090,132 de novo gene families, which accounted for 95% of all plasmid genes (Fig. 1b and Supplementary Fig. 1a) and constituted the primary data for the training of PlasX (Fig. 1, Supplementary Fig. 1b,c and Supplementary Information).

PlasX is a logistic regression that assigns a positive or negative coefficient to gene families that are likely to originate from sequences that are of plasmid or non-plasmid origin (Fig. 1c). The algorithm predicts whether or not a given sequence is a plasmid by considering the coefficients of all gene families in the sequence, and assigns a score to each prediction that ranges between zero and one; a prediction score of >0.5 suggests that the sequence is more likely to be a plasmid than not. To benchmark PlasX, we compared its performance with three state-of-the-art algorithms—PlasClass^20^, PPR-Meta^32^ and Platon^33^—using a cross-validation strategy in which we trained models on a subset of non-redundant reference sequences and evaluated their performance on the remaining sequences that were not used for training (Supplementary Information). PlasX achieved the highest area under the precision–recall curve (AUCPR = 0.70), which was a substantial improvement compared with the next-best method Platon (AUCPR = 0.23; Fig. 1d). We then conducted a test using 21,012 plasmids that were recently included in a large plasmid database (PLSDB)^34^. PlasX correctly identified 81.5% (17,128) of these sequences as plasmids, whereas Platon, the next-best method in our cross-validation tests, only predicted 37.4% (7,860) as plasmids in its most sensitive mode (Supplementary Table 2). We further evaluated the performance of PlasX and other methods on a recently characterized plasmid of Wolbachia, pWCP^35^. PlasX was able to predict pWCP as a plasmid (score = 0.73), whereas none of the other methods in our tests recognized pWCP as a plasmid (Supplementary Table 3). Finally, as an additional benchmarking step to determine the ability of PlasX to distinguish plasmids from other mobile genetic elements, we ran PlasX on all integrative and conjugative element (ICE) sequences from the ICEberg database^36^ (*n* = 552) and all prophage sequences from the National Center for Biotechnology Information (NCBI) viral database (*n* = 445). PlasX correctly classified 92.2% of ICEs (Supplementary Table 4) and 93.2% of the NCBI viral database (Supplementary Table 5) as not plasmids. Platon could also distinguish prophages from plasmids with high accuracy (99.6%) but its classification accuracy was much lower compared with PlasX for ICEs, as Platon classified 37.1% of ICEs as plasmids.

The improved efficiency of PlasX comes from its reliance on de novo gene families rather than sequence features or gene functions alone. Genes in microbial sequence collections often cannot be annotated to a known function, and a known function often groups together genes with large sequence differences. By partitioning genes into homologous groups, de novo gene families both increase the fraction of usable input data for training and increase the resolution of the resulting units that improve training and prediction. For instance, the Pfam PF10609 is a broad family of genes related to *parA*, a gene that drives the partitioning of not only chromosomes^37^ but also plasmids^38^ during cell division. As genes that resolve to this function are found on 35% of plasmids and 95% of chromosomes, it has no ability to distinguish plasmids and chromosomes (coefficient of −0.023). However, PF10609 in our dataset could be subdivided into two de novo gene families, one of which was plasmid-specific (coefficient of +0.455) and the other chromosome-specific (coefficient of −0.198). A clear divergence of plasmids and chromosomes into monophyletic groups was indeed observed for the gene sequences in the two de novo gene families of PF10609 (Fig. 1f,g). Furthermore, 35.5% (398,174) of the de novo gene families did not resolve to a known function, despite that many had highly positive coefficients. In fact, 12,076 of gene families with unknown functions had coefficients of >0.1, and of the 200 gene families with the highest coefficients, 129 did not have any functional annotation. Our survey of gene families (Supplementary Table 6) highlights the difficulty of using functional annotations alone to infer the importance of a gene family in plasmid recognition. Although some gene families included keywords such as ‘plasmid’, ‘replication’ or ‘conjugation’ in their functional descriptions to offer naive confirmations for plasmid relevance, most others were difficult to immediately associate with plasmids. For instance, the gene family with the 17th highest PlasX coefficient of 1.678 in our list was a family of lipoproteins (PF05714). Its annotation, ‘*Borrelia burgdorferi* virulent strain associated lipoprotein’, does not explicitly associate it with plasmids. However, it occurred in 168 plasmids and only two chromosomes in our training data and has been studied previously for conferring virulence in plasmids^39,40^, which suggests that PlasX-assigned coefficients offer an effective means to identify key gene families to recognize plasmids.

Overall, these results show that PlasX performs better than state-of-the-art plasmid prediction approaches in cross-validation tests, is able to recognize plasmids that are not present in existing databases and is less likely to confuse other mobile genetic elements with plasmids.

### PlasX unveils diverse plasmids of the human gut

**Fig. 2:**
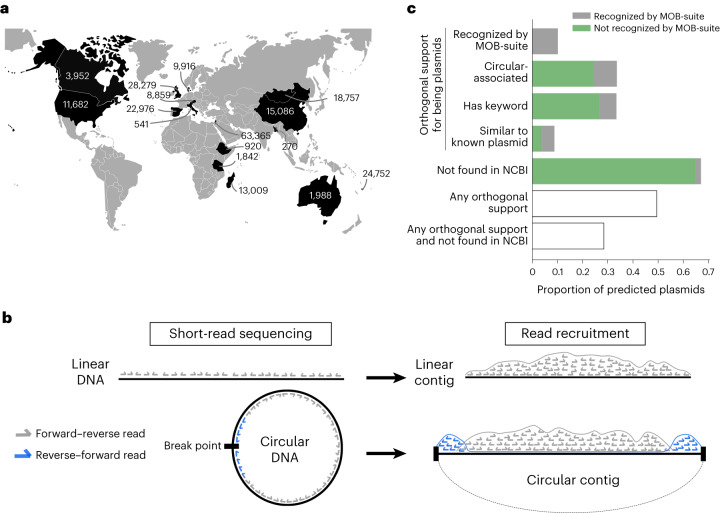
Plasmid prediction from metagenomes. **a**, Number of plasmids predicted from different countries. **b**, Diagram of paired-end reads mapping to a linear contig versus a circular contig. Linear contigs have forward–reverse reads only, whereas circular contigs also have reverse–forward reads concentrated on the ends due to an artefact in contig assembly. **c**, Orthogonal support for the 100,719 non-fragment predictions and their presence in NCBI. 10.1% of predictions were recognized as plasmids by MOB-suite. Among the predictions that were not recognized by MOB-suite, we found that 24,689 (24.5% of the 100,719 non-fragment predictions) were circular-associated sequences, 26,921 (26.7%) were ‘keyword-recognizable’ (as they contained a COG or Pfam function with the word plasmid or conjugation), 3,996 (4.0%) were highly similar to a known plasmid sequence in NCBI and 65,117 (64.7%) had no hits to any sequence in NCBI. As these different subsets of plasmids partially overlap, we took their union to find that 49,739 (49.4%) of predictions had some orthogonal support for being a plasmid, by MOB-suite or any of the other three types of analyses, and 28,658 (28.5%) had such support and were not found in NCBI.

Next, we applied PlasX to survey naturally occurring plasmids in the human gut microbiome, an environment that harbours a diverse range of microorganisms and mobile genetic elements^41^. For this, we assembled 36 million contigs from 1,782 human gut metagenomes, spanning culturally and geographically distinct human populations (Supplementary Table 7). Running PlasX on these data resulted in a total of 226,194 predicted plasmids with a score of >0.5 (Fig. 2a, Supplementary Fig. 2a and Supplementary Table 8). Our predictions spanned a wide range of lengths, including 135 sequences that were longer than 100 kbp, but they were generally shorter than reference plasmids with a median length of 2.6 versus 53.3 kbp, respectively (Supplementary Fig. 2b). Part of this discrepancy is most probably due to the fragmented nature of assembled sequences from metagenomes. The median length of the entire set of contigs was 2.1 kbp and only 50,310 (0.14%) contigs were longer than 100 kbp. To minimize the impact of assembly fragments in our results, we removed predictions that did not seem to be circular and, at the same time, seemed to be fragments of more complete predictions in our collection. This filter left us with 100,719 predictions for downstream analyses (Methods and Supplementary Fig. 3). Although this filtered set inevitably contains fragmented plasmids due to the nature of the input data, hereafter we refer to them as ‘plasmids’ for practical reasons.

To determine the circularity of plasmid sequences in metagenomes, we analysed the orientation of mapped metagenomic paired-end reads (Fig. 2b). With this approach we found that 19,652 plasmid sequences were circular, and we designated them as high-confidence plasmids for downstream analyses. Circular plasmids had a median length of 4.4 kbp and included sequences that were longer than 25 (*n* = 854), 50 (*n* = 378) and 100 kbp (*n* = 47). An additional 14,151 sequences were not circular themselves but were highly similar to a circular sequence. Together, these two types of sequences defined a set of ‘circular-associated’ sequences representing 33.6% (33,803/100,719) of the predictions. Multiple factors can explain the lack of signal for circularity for the remaining plasmids, including insufficient sequencing depth to observe a sufficient number of reverse–forward pairs, fragmented contigs, or the non-circular nature of some plasmids that occur linearly^42^ or those that are integrated into chromosomes^2^.

Beyond circularity, confirming in silico whether a sequence represents a plasmid is a remarkable challenge. Although single-copy core genes have been used to assess the completeness of non-plasmid and non-viral genomes assembled from metagenomes^13^, our understanding of the canonical features of plasmids is limited to a relatively small set of well-studied genes that are primarily derived from plasmids of model organisms in culture^24,25^. For instance, MOB-suite^25^ identified canonical features for plasmid replication and conjugation in only 61% of the reference plasmid subtypes we used to train PlasX, which reveals the limits of conventional approaches to identify plasmid features and survey previously undescribed plasmids (Supplementary Table 1). Indeed, MOB-suite identified canonical features in only 10.1% of our predictions (Supplementary Table 8). Given this narrow sensitivity, we developed orthogonal data-driven strategies to increase confidence in our predictions (Supplementary Information). We found that 49.4% (49,739) of predictions had orthogonal support for being a plasmid—by MOB-suite or other metrics (Fig. 2c)—and 28.5% (28,658) had such support but were not in the NCBI database (Supplementary Table 8). Overall, these findings suggest that our collection of predicted plasmids include not only sequences that match known plasmids but also uncharacterized ones that can further advance our ability to infer the gene pool and ecology of naturally occurring plasmids.

Although conducting experiments is the most reliable strategy for validation, the labour-intensive and low-throughput nature of such investigations represent a substantial limit to their scale. Nevertheless, to experimentally validate at least some of our metagenome-derived predictions as true plasmids of the human gut, we developed a pipeline to identify predictions that (1) are present in human gut microbial isolates, (2) are circular in those isolates and (3) can be naturally transferred to other microorganisms. First, we detected 127 of our predicted plasmids in 14 *Bacteroides* isolate genomes that we sequenced in a previous study^43^ (Supplementary Fig. 4). For two of these plasmids, pFIJ0137_1 and pENG0187_1, we performed additional short-read and long-read sequencing to obtain complete plasmid genomes and confirm their circular configuration (Supplementary Fig. 5). Finally, we demonstrated the ability of pFIJ0137_1 to transfer as a plasmid and confer antibiotic resistance from one *Bacteroides fragilis* host to another (Methods and Supplementary Fig. 4). Although not comprehensive, these experimental results show that PlasX is able to predict plasmids that have canonical features of being extrachromosomal, circular and transmissible between cells.

Overall, our survey of individual assemblies of human gut metagenomes using PlasX resulted in 100,719 plasmid sequences for in-depth characterization.

### Predicted plasmids are prevalent and reflect human biogeography

**Fig. 3:**
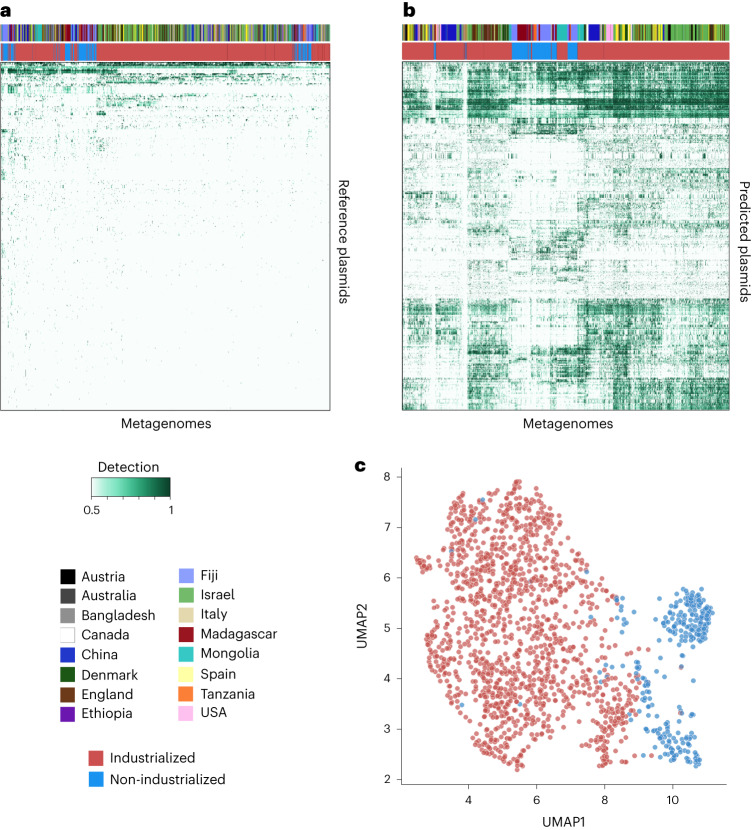
Global plasmid ecology. **a**, Read recruitment of human gut metagenomes to 11,121 non-redundant reference plasmids. The heat map shows the 338 plasmids that are present in at least one metagenome (≥0.95 detection). **b**, Read recruitment to 68,350 non-redundant predicted plasmids. The heat map shows the 1,000 most prevalent plasmids that are present in at least one metagenome and have a PlasX score of ≥0.75. **a**,**b**, The country of origin (top row) and lifestyle (industrialized or non-industrialized; second row) are indicated. The legends in **a** apply to both panels. **c**, Clustering of metagenomes based on the predicted plasmids that are present using the UMAP dimensionality reduction method^86^. Metagenomes from industrialized and non-industrialized populations are coloured red and blue, respectively.

Next, we sought to investigate the ecology of plasmids across human populations by creating a non-redundant collection of all plasmids and using metagenomic read recruitment to quantify their distribution across individuals. The de-replication step resulted in 11,121 non-redundant reference plasmids and 68,350 non-redundant predicted plasmids. Recruitment of short reads from the 1,782 globally distributed human gut metagenomes showed that only 1.9% of reference plasmids were present in at least two individuals in our dataset, revealing the limited ecological relevance of reference plasmids to naturally occurring gut microbial communities (Fig. 3a). Such a weak detection of reference plasmids in the human gut is probably due to the heavy representation of human gut-associated plasmids in public databases that originate from a relatively small number of human pathogens that are not typically abundant in healthy humans. The reference plasmids did include those that were extremely prevalent across human metagenomes, such as pBI143, a cryptic plasmid that was present in 52% of the gut metagenomes in our dataset, which we investigated in depth elsewhere^44^. However, the predicted plasmids were much more prevalent across human populations in general (Fig. 3b); 63.1% of the predicted plasmids were present in at least two individuals (Supplementary Fig. 2d). In fact, 99.7% of all plasmids that occurred in 100 or more individuals were predicted plasmids.

Due to their increased representation in naturally occurring gut microbiomes, the plasmids we predicted from metagenomes better capture the biogeography and lifestyles of human populations compared with reference plasmids. The organization of metagenomes based on their plasmid content showed that only 50.2% of individuals were placed next to someone from the same country based on reference plasmids (Fig. 3a). This percentage increased to 74.0% with plasmids from metagenomes (Fig. 3b). Furthermore, their distribution distinguished between individuals from industrialized and non-industrialized countries (Fig. 3c) and revealed country-specific clustering of metagenomes (Supplementary Fig. 6).

These results parallel other studies that found associations linking the gut microbial taxonomy with the geography and lifestyles of human populations^45^, and thus they lead to an important question: if plasmids and microbial taxa are each correlated with human geography, is microbial taxonomy also correlated with plasmid distribution patterns? On the one hand, it would be conceivable to expect a strong correlation between the two as plasmids rely on host machinery for replication and thus their presence in an environment depends on the presence of a suitable microbial taxon. On the other hand, such associations may be weak or even non-existent for two reasons: (1) some plasmids are known to have a broad host range^46^ and thus, their presence in a given environment might not be consistently associated with the presence of a single species or even higher taxonomic category such as a genus or phylum, and (2) plasmids can be gained or lost as a function of environmental pressures, such that nearly identical microorganisms can differ by the presence or absence of a plasmid or in the number of plasmid copies. By taking advantage of a large number of plasmids that are representative of human biogeography, we examined the ecological associations between plasmid distribution patterns and microbial taxonomy to determine to what extent plasmid ecology can be explained by microbial taxonomy.

### Plasmid ecology is not explained by microbial taxonomy

For every plasmid, we inferred its most probable host as the taxonomic group that had the most similar ecological distribution (Methods). Although some predicted plasmids had a high ecological similarity with their best matching taxonomic group, the vast majority of predicted plasmids had low similarity scores (median correlation = 0.04, median Jaccard = 0.21; Methods and Supplementary Fig. 7a,b). We also observed low similarity scores even for reference plasmids that are isolated from a defined microbial host (Supplementary Fig. 7c,d). For example, the plasmid pDOJH10S and its cognate host, *Bifidobacterium longum*, were present together in ten metagenomes; however, in 27 metagenomes we only found the plasmid and in 69 metagenomes we only found the host (Supplementary Fig. 7e). A more careful consideration of the detection patterns we recovered from metagenomic read recruitment also showed low overlap between host and plasmid sequences across samples, with a Jaccard index of 0.40 (Supplementary Fig. 7f and Supplementary Table 13). The weak correlations between plasmid distribution patterns and microbial taxonomy suggest that plasmids are a highly complex and dynamic feature of microbiomes (Fig. 3a,b and Supplementary Fig. 2d), forming an ecological dimension that can stratify human populations (Fig. 3c and Supplementary Fig. 6) in ways that cannot be explained by microbial taxonomy alone (Supplementary Fig. 7). Although high-throughput analyses of human gut microbiomes often focus on taxonomic features, it has been challenging to find significant or reproducible taxonomic associations that distinguish health and disease states^47^. As plasmids often carry key determinants for survival in an environment, a complete understanding of the microbial ecology of health and disease states probably requires the inclusion of insights into plasmid ecology, which requires not only the recovery of naturally occurring plasmids through strategies such as PlasX but also the characterization of their gene pool in an evolutionary framework, which we aimed to do next using MobMess.

### Plasmid systems elucidate backbone versus cargo genes

Our large collection of naturally occurring plasmids provides a unique opportunity to study evolutionary patterns in the human gut plasmidome. Due to frequent genetic rearrangements, a hallmark of plasmid evolution is the reuse of a backbone complemented with variable cargo/accessory genes^6,8,10,48^. Plasmid backbones—which so far have been characterized based on nucleotide identity^6,7,49^, gene similarity^10^ or gene annotations^50–52^—typically encode machinery necessary for plasmid maintenance, and the cargo genes represent additional genetic content, such as antibiotic resistance or other fitness-determining functions.

**Fig. 4:**
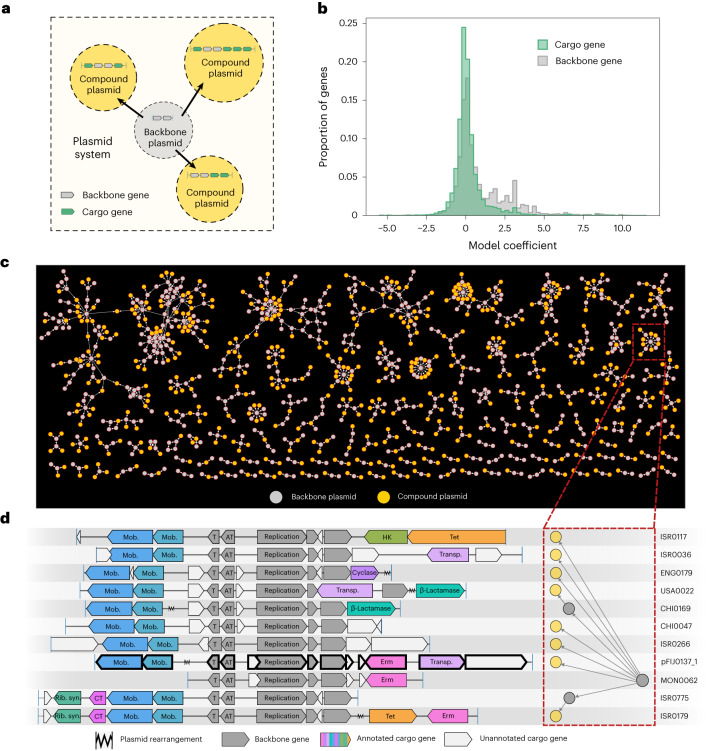
Identification of plasmid systems. **a**, Network diagram of a plasmid system. **b**, Distribution of model coefficients for backbone and cargo genes in the non-redundant set of 68,350 predicted plasmids. We excluded genes that lacked gene family annotations and thus have a coefficient of zero by default. We also excluded genes that were labelled as backbone with respect to some systems but cargo in others. **c**, Network of all plasmid systems that contain ≥3 non-redundant and high-confidence plasmids. Only these types of plasmids are shown. **d**, Genetic architecture of plasmids in PS486, encased by a red box in **c**. Two plasmids in **c** were excluded and the plasmid outlined in bold marks pFIJ1037_1, a plasmid we isolated and transferred between *B. fragilis* strains in this study. The system’s backbone (assembled from metagenome MON0062) encodes five backbone genes (coloured grey). Rib. syn., riboflavin biosynthesis; CT, conjugative transfer; mob., mobilization; T, toxin; AT, antitoxin; tet, tetracycline resistance; erm, erythromycin resistance; transp., transposon; and HK, histidine kinase.

Here we designed a network-partitioning algorithm, MobMess, to study backbone structures in any collection of plasmid sequences at scale (Supplementary Fig. 8). Briefly, MobMess first calculates pairwise alignments across all plasmids to build an initial sequence-similarity network in which a directed edge represents the containment of one plasmid within another, defined by ≥90% sequence identity and ≥90% coverage of the smaller plasmid. We found that these thresholds represent a natural divide between related and distant plasmids, as we observed a ‘valley’ at these thresholds in the distribution of pairwise average nucleotide identities between all predicted plasmids (Supplementary Information and Supplementary Fig. 9). Next, MobMess recognizes and collapses redundancy between plasmids and analyses patterns of connectivity in the network to identify ‘backbone plasmids’ that satisfy two criteria: (1) the backbone plasmid must be a circular element, inferred here by paired-end orientation (Fig. 2b), to ensure that it is not an assembly fragment and can replicate as an independent element and (2) a backbone plasmid must be found as a subsequence within one or more ‘compound plasmids’. These compound plasmids are composed of the backbone and additional cargo, indicating the ability to acquire or lose genes. Here we define a backbone and its compound plasmids as an evolutionary unit called a plasmid system (Fig. 4a).

MobMess differs in multiple ways from recently described clustering approaches that have been applied to plasmid sequences^53,54^, which have only been tested on reference plasmids that are complete. We designed MobMess to handle complex scenarios by distinguishing between fragmented and complete (circular) plasmids, a strategy that renders MobMess more suitable to work with complex datasets such as metagenomic assemblies, where sequence fragmentation is a common problem. Tracking the containment of smaller plasmids within larger ones is also a severe computational challenge that is overlooked by previous approaches (Supplementary Information). MobMess can distinguish whether a group of plasmids simply share a level of homology through partial alignments or a more nuanced evolutionary relationship through a common backbone that can replicate independently (Supplementary Fig. 10). In this way MobMess minimizes spurious connections between distinct plasmid entities while maximizing its inference of evolutionarily cohesive groups as plasmid systems (Supplementary Fig. 11 and Supplementary Information).

This definition of plasmid systems facilitates analyses of plasmid backbone versus cargo content as well as their ecology when used in conjunction with metagenomic data, much in the same way that pangenomes enable studies of core versus accessory gene content in microbial genomes. However, plasmid systems are a specific case of pangenomics as it is unlikely to find a naturally occurring microbial genome composed only of core genes. In contrast, backbone plasmids represent a minimal entity that can propagate using only backbone genes. With its algorithmic considerations, MobMess provides an automated framework and vocabulary to explore the concept of plasmid systems across different studies and datasets.

### Plasmid systems carry a wide repertoire of cargo functions

Our application of MobMess to the plasmid sequences predicted by PlasX from human gut metagenomes resulted in a total of 1,169 plasmid systems. Plasmid systems captured a small fraction of the genetic diversity among non-redundant plasmids (6.5%, 4,424/68,350); however, they captured a large fraction of all circular plasmid contigs (72.7%, 14,285/19,652; Methods and Supplementary Table 9) and plasmids that were part of a system tended to be longer than plasmids that were not part of any system (Supplementary Table 10). Due to our stringent criteria for the inclusion of plasmids in plasmids systems, MobMess identifies reliable plasmids with multiple representatives in a given dataset independently of the initial confidence scores assigned by PlasX. For instance, of all plasmids with scores between 0.5 and 0.9, MobMess placed 16,663 in plasmid systems, which retrospectively suggests that a strict cutoff on PlasX prediction scores (such as >0.9) will remove many genuine plasmids from downstream analyses.

Plasmid systems were highly heterogeneous in their genetic complexity. Thirty-seven plasmid systems contained sequences that could be classified into seven different plasmid incompatibility types (Inc11, Inc18, IncFIB, IncFIC, IncI-ɣ/K1, IncK2/Z and IncW; Supplementary Table 9). Furthermore, 602 plasmid systems contained at least two non-redundant compound plasmids, with the largest system containing 168 non-redundant compound plasmids (Fig. 4c). For example, pFIJ1037_1, one of the plasmids we isolated and transferred between *B. fragilis* strains, was part of PS486, a system containing 24 non-redundant plasmids and found across a total of 127 metagenomes. The PS486 backbone consists of a replication protein and a toxin–antitoxin system, and the cargo genes include β-lactamases, erythromycin resistance, tetracycline resistance and riboflavin biosynthesis (Fig. 4d and Supplementary Table 11).

To understand how much genetic content is typically conserved or variable in a plasmid system, we calculated the percentage of genes on compound plasmids that were backbone genes versus cargo genes (Methods). Compound plasmids contained a wide range of cargo gene percentages occurring anywhere between 0% and 100%, with a median value of 40% (Supplementary Fig. 12). Conversely, the median backbone percentage was 60%. PlasX often assigned higher model coefficients to backbone genes in the non-redundant set of predicted plasmids, suggesting that these genes define the ‘essence’ of a plasmid by encoding essential functions that promote the ability of a plasmid to exist as a distinct element from the chromosome, such as the genes for plasmid replication (*repA*; PF01051) and mobilization (*mobA*; PF03432; Fig. 4b). In contrast, PlasX assigned lower coefficients to cargo genes, suggesting that they encode functions that are not universally essential but important for specific niches, such as nitrogen reductase (*nifH*; PF00142) and membrane transport (*ompA;* PF00691). We found that 24.1% (2,169/8,995) of backbone genes, versus 13.4% (3,229/24,168) of cargo genes, encoded COG and Pfam functions with descriptions related to plasmid replication, transfer and maintenance (Supplementary Information).

**Fig. 5:**
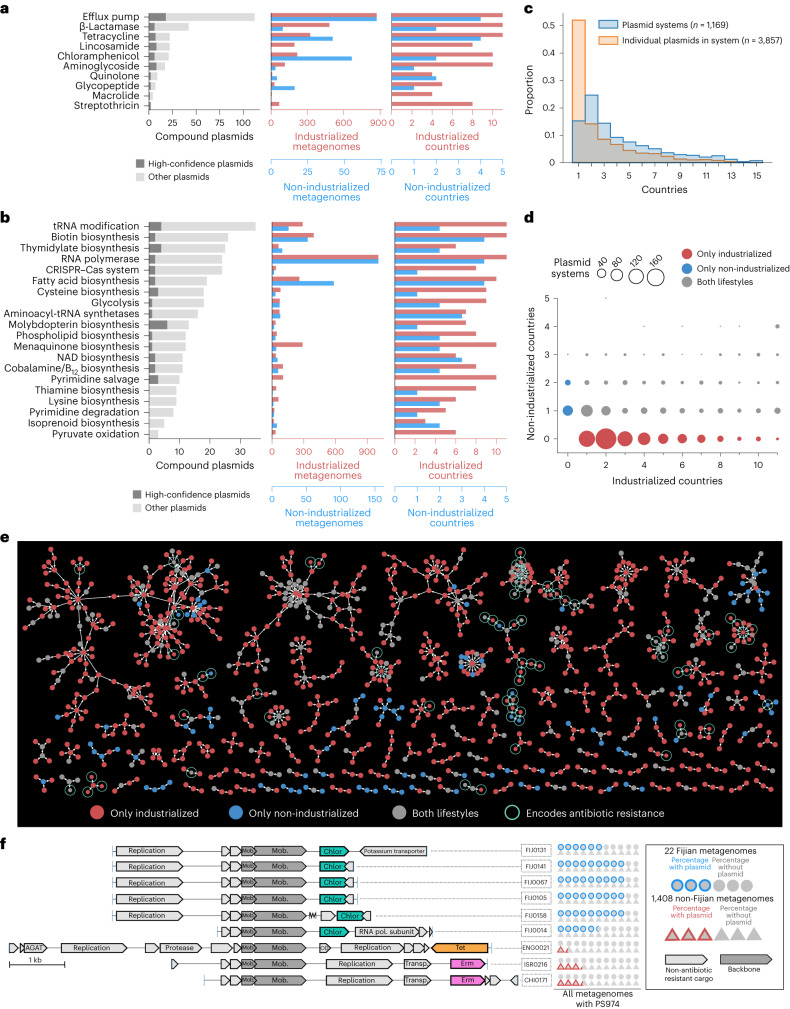
Functional and ecological variation of plasmid systems. **a**,**b**, Number of compound plasmids that encode antibiotic resistance (**a**) and COG pathways (**b**) in cargo genes. Also shown are the numbers of metagenomes and countries that contain those plasmids. **b**, The 20 COG pathways with the highest number of compound plasmids. To avoid redundancy with **a**, we excluded COG pathways that occur in cargo genes encoding antibiotic resistance. **c**, Prevalence of plasmid systems versus the individual plasmids in those systems. **d**, Distribution of plasmid systems according to the number of industrialized and non-industrialized countries they are found in. **e**, Recolouring of the network of plasmid systems shown in Fig. 4c. **f**, Compound plasmids from PS974 that encode for resistance to chloramphenicol, tetracycline or erythromycin. Six of the nine plasmids are circular. Chlor, chloramphenicol resistance; tet, tetracycline resistance; erm, erythromycin resistance; AGAT, aminoglycoside adenylyltransferase; and OD, oxaloacetate decarboxylase. PS974 is found in 22 Fijian and 1,408 non-Fijian metagenomes. The pictogram (right) represents these metagenomes using circles (Fijian) and triangles (non-Fijian), and those with coloured outlines represent the proportion of the corresponding metagenomes that contain the plasmid.

The most frequent type of function encoded on cargo genes was antibiotic resistance, including efflux pumps, which can provide general resistance to multiple antibiotics, and genes targeting specific classes of antibiotics, such as glycopeptides and β-lactams (Fig. 5a). This large-scale observation is consistent with numerous examples of known plasmids encoding resistance and further illustrates how the widespread presence of these plasmids poses a public health threat^55,56^. Other highly prevalent cargo functions included a wide diversity of cellular and metabolic pathways defined in the COG (Fig. 5b) and Kyoto Encyclopedia of Genes and Genomes (KEGG) databases (Supplementary Fig. 13). The most enriched among these was transfer RNA modification, encoded in 35 compound plasmids within different systems. For example, the globally prevalent system PS1110 (present in 739 metagenomes) contained 291 compound plasmids (27 non-redundant), three of which encoded an enzyme that performs tRNA Gm18 2′-*O*-methylation (COG0566) and were collectively present in 498 metagenomes (Supplementary Fig. 14 and Supplementary Table 11). This enzyme is thought to reduce the immunostimulatory nature of bacterial tRNA, which is detected by Toll-like receptors (TLR7) of the mammalian innate immune system^57,58^. Although plasmids in some pathogens are known to facilitate bacterial evasion of the mammalian immune system by regulating surface proteins^59^, the overwhelming prevalence of tRNA modification enzymes in our data suggests the likely presence of a previously unappreciated role for plasmids to increase the fitness of their bacterial hosts against the surveillance of the human immune system.

Overall, these results show that the plasmid systems we were able to identify from the human gut using MobMess represent evolutionarily cohesive units with the enrichment of different classes of functions in the backbone and cargo gene pools. Functional annotations further suggest that whereas the conserved pool of backbone genes can yield insights into plasmid compatibility and maintenance, the dynamic pool of cargo genes could serve as a means to identify genetic determinants of fitness that respond to particular environmental conditions.

### Plasmid cargo are genes adapted to specific environments

Given the highly heterogeneous biogeography of individual plasmids (Fig. 3b) and their organization into their country of origin (Supplementary Fig. 6), we next investigated whether the higher-order evolutionary units described by plasmid systems simply consisted of plasmids with similar distribution patterns or spanned larger geographical regions with individual plasmids of distinct ecology. Our analysis of plasmids and plasmid systems across metagenomes showed that although individual plasmids were often present in a single country, plasmid systems frequently spanned multiple countries (Fig. 5c and Supplementary Table 9). Of the 2,005 individual plasmids that were unique to a single country, 1,794 (89.5%) were part of more geographically diverse plasmid systems that were present in at least two countries. In fact, 84% (982/1,169) of the plasmid systems in our dataset were present in at least two countries and we found that nine plasmid systems were present in as many as 15 of the 16 countries (Supplementary Table 9), suggesting that cargo genes that were likely selected for in different regions stemmed from conserved backbone structures.

The broad ecological distribution patterns of plasmid systems compared with the country-specific distribution patterns of plasmids they describe presents a unique opportunity to gain insights into environmental pressures that drive the composition of cargo genes within individual plasmid systems. To investigate this further, we focused on plasmid systems that spanned industrialized and non-industrialized countries, which were largely separated by the distribution of individual plasmids (Fig. 3c). Many plasmids systems were exclusive to either industrialized or non-industrialized countries; however, 396 were present in both (Fig. 5d). Antibiotic usage is a well-understood environmental pressure that often requires microorganisms to maintain plasmids with antibiotic-resistance genes^60^. In our data the evolution of antibiotic resistance in a plasmid system coincided with the ecological variation of compound plasmids in the system. Specifically, we identified 24 high-confidence compound plasmids that encoded antibiotic resistance in cargo genes and were exclusively present in either non-industrialized or industrialized countries (Fig. 5e). Among the non-industrialized metagenomes, one of the most common types of antibiotic resistance is chloramphenicol resistance (Fig. 5a). For instance, the plasmid system PS974 contained 97 non-redundant plasmids; however, this system possessed chloramphenicol resistance (conferred via an acetyltransferase) only in plasmids from Fiji (Fig. 5f and Supplementary Table 11). When we searched for these resistance plasmids across the global set of 1,430 metagenomes that contain PS974, we found them in 19/22 Fijian metagenomes but only in 1/1,408 non-Fijian metagenomes (*P* = 1.1 × 10^−13^, Fisher’s exact test; Fig. 5f, pictogram). Chloramphenicol is routinely prescribed in Fiji to treat eye infections, central nervous system infections, periodontitis, shigellosis, typhoid and paratyphoid fevers, and diabetic foot infections but it is rarely used in North America and Europe^61–63^. Strikingly, PS974 also contained compound plasmids that carry tetracycline resistance (171/1,408 metagenomes) or erythromycin resistance (429/1,408 metagenomes), yet these plasmids only occurred in individuals from China, Israel and the United Kingdom (Fig. 5f). Matching sequences to these plasmids on the NCBI databases suggested that their possible microbial hosts include populations in the phylum Firmicutes, such as *Blautia hydrogenotrophica*. However, the distribution patterns of plasmids in PS974 did not match any microbial taxa (highest Jaccard index = 0.37 across all plasmid-taxon comparisons). Our observations here suggest that despite sharing a common backbone, compound plasmids in PS974 give access to different antibiotic-resistance genes and their ecology is defined by lifestyle-specific usage of antibiotics.

Overall, these data show that plasmid systems can reveal cargo genes that serve as probable determinants of fitness given known environmental pressures. Conversely, plasmid systems can also support hypothesis generation through insights into selective pressures given the known functions that differentially occur across environments as cargo, providing a biologically meaningful computational framework to study plasmid ecology and evolution at scale.

## Discussion

Plasmids are found in nearly every microbial ecosystem, yet the computational challenges associated with their de novo identification have made it difficult for microbiologists to routinely survey plasmids and define evolutionarily cohesive units to describe plasmid diversity in complex environmental samples. PlasX and other plasmid recognition systems^19,20,22–24,33^, along with MobMess to characterize plasmid systems, present a powerful roadmap for a detailed characterization of naturally occurring plasmids at scale. Although our study focused on the human gut microbiome, we designed PlasX and MobMess using a broad collection of reference sequences so that they can be applied to study any environment, and with the flexibility to include additional training sequences to improve accuracy. These methods provide a complementary approach to frequently used state-of-the-art workflows to study the taxonomic composition or functional potential of environmental or host-associated microbiomes through amplicon sequences or metagenomes.

Historically, plasmids and other genetic elements have been characterized on the basis of qualitative properties and descriptions. Early applications of machine-learning approaches to predict plasmids employed a small number of marker genes, which limited the recognition of plasmids that lacked recognized markers. The effect of this shortcoming is clear, as many of the genuine plasmids contained no such markers (Fig. 2c). Another popular strategy to recognize plasmids has been to employ *k*-mers, but such algorithms will also miss plasmids that have low sequence similarity to those that are described in public databases (Fig. 1d). By relying on gene families, PlasX presents an unbiased addition to our bioinformatics toolkit to predict plasmids. However, although both our quantitative and qualitative surveys showed that PlasX surpasses the performance of state-of-the-art algorithms to identify plasmids, it is essential for researchers to take into consideration that predicted sequences will contain both false positives and false negatives. Plasmids can be difficult to distinguish from other mobile or integrated genetic elements as they share common features, including that they are extrachromosomal^64^, facilitate horizontal gene transfer^65,66^ and encode traditional core functions such as replication and mobilization^25,67^. The accuracy of PlasX is also tied to the underlying training set of reference plasmids and chromosomes; over- or under-representation of certain types of sequences can bias the model and limit generalizability.

The confidence scores PlasX assigns to each prediction may serve as a means to adjust the amount of noise in the prediction results; however, researchers must consider the trade-off between sensitivity (that is, capturing a higher fraction of true plasmids) versus specificity (that is, reducing the number of falsely predicted plasmids) while setting a cutoff. The trade-off between sensitivity and specificity was most visible in our cross-validation analyses, where a threshold of >0.5 lies at an inflection point in the precision–recall curve in Fig. 1d (with a precision of 0.850 and recall of 0.500). This threshold was also sufficient at distinguishing ICEs and prophages from plasmids. The application of a stricter threshold of >0.9 increases precision by 13% (to 0.920) but it also decreases recall by 44% (to 0.280). As our understanding of plasmid diversity in metagenomes is greatly underdeveloped, here we decided to apply a threshold of >0.5 to provide a reasonable balance between precision and recall for our study and to include many potentially uncharacterized plasmids in our results. A good example of this is the long-missed *Wolbachia* plasmid^35^, which has a score of 0.73. At the same time, stricter thresholds (such as >0.9) or other filters such as circularity may be more appropriate in future work, where a higher precision of predicted plasmids (fewer false positives) is of higher priority.

Given the dynamism of plasmids, organizing them into evolutionarily cohesive groups is a formidable challenge. Previous computational methods for organizing plasmids have relied on average nucleotide identity to represent the whole-sequence similarity between plasmids but, although computationally tractable, this single statistic misses other evolutionary dimensions that relate sequences. To address these issues, we developed MobMess, which resolves the containment of plasmid sequences within one another. This methodological advance enabled us to identify plasmid systems, revealing the great extent to which plasmids in complex ecosystems are not static entities but actively evolving in response to the environment. Plasmid malleability is a desirable property in bioengineering and has often motivated the repurposing of naturally occurring plasmids into major tools for genetically modifying organisms. In this vein, we propose computational identification of plasmid systems as an attractive approach to expand the toolkit of available plasmids for genetic engineering, particularly if they are found in isolates that currently lack tools to make them genetically tractable. Plasmid systems, which manifest in many distinct forms across multiple human populations, excel at incorporating additional functions and propagating across a wide range of natural environments and may behave similarly in laboratory settings.

An overarching implication of our findings is that high-throughput recognition and characterization of plasmids in microbiome studies are necessary for more complete insights into the ecology of naturally occurring microbial systems.

## Methods

### Compiling and annotating a reference set of plasmids and chromosomes

We obtained a list of 16,168 plasmids from the 2019_03_05 version of PLSDB^68^. We also downloaded the entire collection of 13,471 complete bacterial genome assemblies from NCBI RefSeq on 26 October 2019, using instructions at https://www.ncbi.nlm.nih.gov/genome/doc/ftpfaq/#allcomplete (ref. ^69^). The RefSeq assemblies contained 26,376 contigs, of which we discarded 11,350 that are also in PLSDB. The reference set of 16,827 plasmids consisted of 16,168 PLSDB contigs as well as 659 contigs from the RefSeq assemblies that were labelled as ‘Plasmid’ in the ‘Assigned-Molecule-Location/Type’ field of the NCBI assembly report. The reference set of chromosomes was the remaining 14,367 RefSeq contigs.

To identify and annotate genes in these sequences, we used the program ‘anvi-run-workflow’ with ‘--workflow contigs’ implemented^70^ in anvi’o^71^ v7.1, which uses Snakemake^72^ to execute previously defined steps (https://merenlab.org/anvio-workflows/) and to generate anvi’o contigs-db files (https://anvio.org/m/contigs-db). These steps include first running Prodigal^73^ to call genes and then running DIAMOND v2.0 (ref. ^74^) and HMMER v3.3 (ref. ^75^) on amino acid sequences to determine gene functions against the amino acid sequences in Clusters of Orthologous Groups (COGs)^30^ and the Hidden Markov Models (HMMs) in the Protein Family Database (Pfams) v32.0 (ref. ^31^), respectively. To minimize noise, we used an *e*-value cutoff of 1 × 10^−10^ for COGs and the default model noise cutoff scores for Pfams.

### Modelling de novo gene families

We inferred de novo gene families by running MMseqs2 (ref. ^76^) v10.6d92c on all amino acid sequences in our reference plasmids and chromosomes. First, we ran ‘mmseqs clusthash’ to collapse identical sequences into a non-redundant set for faster execution of the next step; the collapsing was inverted at the end to annotate all genes. Next, we ran ‘mmseqs cluster’ to calculate pairwise alignments and then cluster genes that are aligned above a minimum sequence identity threshold (parameter ‘--min-seq-id’). We ran this program multiple times with different thresholds (0.9, 0.8, 0.7, 0.6, 0.5, 0.4, 0.3, 0.25, 0.2, 0.15, 0.1 and 0.05) to infer a wide range of possible families. Families from different thresholds can be redundant, so we merged nested families—that is, if family X contains all genes in family Y, then we keep X and discard Y. We also discarded any family that contained only one gene. In theory, families inferred from a higher threshold (for example, 0.9) should always nest within a family inferred from a lower threshold (for example, 0.05) such that we would discard all families from higher thresholds. However, in practice, families do not always nest within each other but only overlap partially. After merging, our final model used the following number of families from each threshold.

**Table:** 

Identity threshold	Number of de novo families
0.05	720,587
0.15	0
0.10	0
0.20	85,042
0.30	71,282
0.25	70,504
0.40	49,106
0.50	23,965
0.60	31,379
0.70	18,837
0.80	10,331
0.90	9,099

In total, our model used 1,090,132 gene families, which annotated 162,783,114 genes. Note that because these gene families can still overlap with each other, a gene may have multiple annotations. This analysis took advantage of MMseqs2’s parallelism, taking approximately 6 h using 256 CPU cores.

We refer to a de novo family as a subfamily if 90% or more of its set of amino acid sequences are also annotated to a specific COG or Pfam. Note that this definition provides a small tolerance such that a subfamily does not need to be a perfect subset of a COG or Pfam. For the example about Pfam PF10609 (Fig. 1f,g), we gathered the 253 amino acid sequences annotated to PF10609 and the subfamily mmseqs_5_1535552. We also gathered the 1,391 sequences annotated to PF10609 and the subfamily mmseqs_70_40217271. We collapsed 100% identical sequences to yield a total collection of 142 and 310 sequences from mmseqs_5_1535552 and mmseqs_70_40217271, respectively. We aligned all of these sequences together using muscle v3.8.1551 (default parameters)^77^ and then constructed a maximum-likelihood phylogenetic tree using IQ-TREE v2.1.2 (parameters -m TEST -bb 1000 -alrt 1000 -T AUTO)^78^. We then rooted the tree using the midpoint method.

### Subtypes and slicing of reference sequences

To group reference sequences into subtypes, we used mash v2.2.2 (ref. ^79^; command ‘mash dist’; sketch size, 100,000; *k*-mer size, 21) to calculate a distance score of 0–1 between every pair of sequences. Next, we created an undirected graph, where sequences are nodes and sequences are connected if their distance is ≤0.1. We defined a ‘subtype’ as one of the 7,326 connected components in the graph. A total of 3,935 subtypes contained only plasmids, 3,355 subtypes contained only chromosomes and 36 subtypes contained both plasmids and chromosomes (Supplementary Table 1).

We sliced reference sequences into slices of 10 kbp by sliding a window of 10 kbp at increments of 5 kbp. The first window starts at the beginning of the sequence and the final window stops at the end of the sequence. For instance, a 23 kbp sequence would be sliced at 0–10, 5–15, 10–20 and 13–23 kbp. A slice was annotated with any gene that was entirely or partly inside the slice. We generated a total of 10,453,279 slices from the reference chromosomes and 343,246 slices from the reference plasmids.

### Assessing model performance in cross-validation

We performed fourfold cross-validation by splitting the set of 10-kbp slices of reference sequences into four random subgroups. We used the sequences in three subgroups to train our model, PlasX, and then we evaluated the performance of the model on the fourth subgroup. We repeated this procedure by changing which subgroups were used for training or evaluation a total of four times. This procedure is a common technique in machine learning, more generally known as ‘*k*-fold validation’ where *k* is the number of subgroupings. In a naive split, we keep all slices from the same reference sequence together in either training or testing data. In an informed split, we keep all slices from the same subtype together.

We assigned weights to the slices of 10 kbp when calculating precision and recall performance (Fig. 1d and Supplementary Fig. 1d,f). Consider the following notation to represent sequences:

$${S}_{i}={\mathrm{sequence}}\,i$$

$${P}_{u}={\mathrm{{the}\,{set}\,{of}\,{plasmid}\,{sequences}\,{in}\,{subtype}}}\,u$$

$${C}_{u}=\mathrm{{the}\,{set}\,{of}\,{chromosome}\,{sequences}\,{in}\,{subtype}}\,u$$

$${D}_{i}^{k}=\mathrm{{window}\,{slice}}\,k\,\mathrm{{of}\,{sequence}}\,i$$

In addition, consider the following notation to represent weights:

$$w\Big({D}_{i}^{k}\Big)=\mathrm{{weight}\,{of}\,{window}\,{slice}}\,k\,\mathrm{{of}\,{sequence}}\,i$$

$$w({S}_{i})=\sum _{k}w\Big({D}_{i}^{k}\Big)=\mathrm{{weight}\,{of}\,{sequence}}\,i$$

$$w({P}_{u})=\sum _{{S}_{i}\in {P}_{u}}w({S}_{i})=\mathrm{{weight}\,{of}\,{plasmid}\,{sequences}\,{in}\,{subtype}}\,u$$

$$w({C}_{u})=\sum _{{S}_{i}\in {C}_{u}}w({S}_{i})=\mathrm{{weight}\,{of}\,{chromosome}\,{sequences}\,{in}\,{subtype}}\,u$$

We defined two different scenarios for assigning weights. Scenario A satisfies the following conditions:

(1) All slices from the same sequence have the same weight

$$\,w\Big({D}_{i}^{s}\Big)=w\Big({D}_{i}^{t}\Big)\quad\,\forall s,t$$

(2) The weight of every sequence is equal to one

$$w({S}_{i})=1\quad\,\forall i$$

Scenario B satisfies the following conditions:

(1) All slices from the same sequence have the same weight

$$\,w\Big({D}_{i}^{s}\Big)=w\Big({D}_{i}^{t}\Big)\quad\forall s,t$$

(2) All plasmid (or chromosome) sequences in the same subtype have equal weight

$$w({S}_{i})=w({S}_{j})\quad\forall i,j,u\,\mathrm{{where}}\,{S}_{i}\in {P}_{u}\,\mathrm{{and}}\,{S}_{j}\in {P}_{u}$$

$$w({S}_{i})=w({S}_{j})\quad\forall i,j,u\,\mathrm{{where}}\,{S}_{i}\in {C}_{u}\,\mathrm{{and}}\,{S}_{j}\in {C}_{u}$$

(3) All subtypes have equal weight

$$w({P}_{u})=w({P}_{v})\quad\forall u,v$$

$$w({C}_{u})=w({C}_{v})\quad\forall u,v$$

(4) The sum of weights across all slices equals the total number of slices

$$\sum _{u}w({P}_{u})={\mathrm{total}}\,{\mathrm{number}}\,{\mathrm{of}}\,{\mathrm{plasmid}}\,{\mathrm{slices}}\,({\mathrm{that}}\,{\mathrm{is}}, 343,246)$$

$$\sum _{u}w({C}_{u})={\mathrm{total}}\,{\mathrm{number}}\,{\mathrm{of}}\,{\mathrm{chromosome}}\,{\mathrm{slices}}\,({\mathrm{that}}\,{\mathrm{is}}, 10,453,279)$$

Each scenario implies a unique assignment of weight values. Scenario A requires that every sequence has the same weight. Importantly, this ensures that long sequences, which have disproportionately more slices, have equal weights to shorter sequences. Scenario B further requires that every subtype has the same weight. Importantly, this ensures that subtypes that contain a disproportionately large number of sequences (for example, subtypes that represent commonly studied bacteria, such as *Escherichia*, *Salmonella* and *Klebsiella*) have equal weight as subtypes with fewer sequences.

We evaluated performance under two different cross-validation and weighting scenarios. Supplementary Fig. 1f shows the result of training models using a ‘naive’ cross-validation split and calculating precision/recall using weights from Scenario A. Figure 1d shows the results of training models using an ‘informed’ cross-validation split and calculating precision/recall using weights from Scenario B. We calculated precision/recall using the function sklearn.metrics.precision_recall_curve from the scikit-learn Python package^80^, with the parameter sample_weight set to the weights of the slices. We calculated AUCPR with the function sklearn.metrics.average_precision_score.

### PlasX implementation

We implemented PlasX as a logistic regression using the SGDClassifier class from scikit-learn^80^. For training and evaluating PlasX, we used 10-kbp slices of the reference sequences to normalize for the fact that chromosomes are generally much longer than plasmids and to improve downstream application of PlasX on sequence collections that may contain a large number of fragmented sequences, such as assembled metagenomes. Regardless of how we evaluated PlasX (Fig. 1d or Supplementary Fig. 1f), we always trained it with weights defined by Scenario B and based on only slices in the training data.

To improve performance, PlasX uses a technique called elastic net regularization, which identifies gene families with redundant or noisy signals and then minimizes the usage of these families by setting their coefficients equal or close to zero. Consequently, only a non-redundant and informative set of gene families can impact predictions by having coefficients far from zero (Supplementary Fig. 1d). To implement elastic net regularization, we performed a grid search of hyperparameters, with the regularization parameter alpha ranging from 1 × 10^−8^ to 1 × 10^−3^ in multiplicative increments of $\sqrt{10}$ and the parameter l1_ratio being 0, 0.25, 0.5, 0.75 or 1.0. For each evaluation scenario (Fig. 1d or Supplementary Fig. 1f), we selected the hyperparameters that produced the best performance. We used the best hyperparameters from the informed cross-validation and the weights defined by Scenario B (alpha = 3.16 × 10^−6^; l1_ratio = 0) to retrain PlasX on all 10-kbp slices and create the final model that we used to predict plasmids from metagenomes.

### Predicting plasmids from metagenomic assemblies

We acquired 1,782 publicly available gut metagenomes that represent 16 countries (Supplementary Information). In this list, we labelled Tanzania, Ethiopia, Bangladesh, Madagascar and Fiji as non-industrialized and the remaining countries as industrialized for downstream analyses. We automated all steps of quality filtering, metagenomic assembly, read recruitment and profiling using snakemake^72^ workflows in anvi’o^81^. The illumina-utils^82^ commands ‘iu-gen-configs’ and ‘iu-filter-quality-minoche’ with the flag ‘--ignore-deflines’ were used to quality filter the raw paired-end reads and each metagenome was assembled individually using IDBA_UD^83^ with default settings and the additional flag ‘--min_contig 1000’ to remove contigs shorter than 1,000 nucleotides. We annotated COGs and Pfams in all assembled contigs using the same procedure as the reference plasmids and chromosomes. To annotate gene families de novo, we first used ‘mmseqs result2profile’ (default parameters) to represent the sequence conservation in each de novo family as a profile. We then used ‘mmseqs search’ (default parameters) to search for profiles across all genes. We kept hits where the alignment coverage was ≥80% of both the gene and the profile and where the alignment identity was at least ≥*X* − 0.05 where *X* is the minimum identity threshold used to originally construct the family (parameter --min-seq-id). For example, if a family was constructed using an identity threshold of 0.8, then we kept hits with an identity of ≥0.75. Using these gene annotations, we ran PlasX to assign a score to every contig. We kept contigs intact, rather than slicing them into 10-kbp windows. Contigs with a score of >0.5 were classified as plasmids.

### Detection and circularity of plasmids across metagenomes

We recruited short reads from our collection of metagenomes using Bowtie 2 v2.0.5 (ref. ^84^). We used the snakemake workflows in anvi’o to automate execution of Bowtie and post-processing to calculate ‘detection’ (that is, the proportion of a sequence that is covered by at least one read). We ran Bowtie 2 using the three following combinations of parameters and input files.

First, to identify circular contigs, we recruited each metagenome’s reads to a fasta file that contained only the contigs assembled from that metagenome. For computational efficiency, we ran Bowtie 2 with its default behaviour to align every read at most once. We then analysed the orientation of paired-end reads (Fig. 2b). During assembly, circular sequences are broken by an artificial breakpoint to represent them as linear contigs. Consequently, DNA sequencing that occurred across this breakpoint will produce paired-end reads that align in a reverse–forward orientation to the ends of the contig. In contrast, if a sequence is not circular, then all paired-end reads are expected to align in a forward–reverse orientation. To illustrate this intuition, suppose the upstream read of a paired-end maps to positions 200–300 of a contig and the downstream read maps to 500–600. If the upstream read maps with a reverse complement strandedness (that is, reverse) and the downstream read maps with the same strandedness as the way the contig is written (that is, forward), then the paired end is in a reverse–forward orientation. In other words, if the contig is written 5′-to-3′, then the upstream read maps 3′-to-5′ and the downstream read maps 5′-to-3′. Inversely, the paired end is in a forward–reverse orientation if the upstream read maps 5′-to-3′ and the downstream read maps 3′-to-5′. Next, we defined the gap (or insert) size of a paired end to be the distance between the closest (or farthest) aligned positions between its two reads. In our example the gap size is 600 − 200 = 400 and the insert size is 500 − 300 = 200. Let *D* be the length of the contig minus three times the median insert size of all forward–reverse paired ends that are aligned to the contig. Finally, we labelled a contig as circular if: (1) its detection was ≥0.95 and (2) it had at least one reverse–forward paired end with a gap that was ≥*D*. This approach of examining reverse–forward paired ends was inspired by ref. ^85^. There were 154,680 contigs that were not predicted to be plasmids but still seemed to be circular; however, these contigs tended to have a smaller number of supporting reverse–forward reads relative to their coverage (Supplementary Fig. 2e), which may indicate that they are other types of mobile elements such as viruses or ICEs that temporarily circularize.

Second, to study the ecological distribution of all plasmids and plasmid systems at the same time, we recruited each metagenome’s reads to a fasta file that contained either the non-redundant set of 68,350 predicted plasmids or the non-redundant set of 11,121 reference plasmids. For computational efficiency, every read was aligned at most once (that is, the default behaviour of Bowtie). A plasmid was considered present in a metagenome if its detection was ≥0.95. To compare metagenomes based on their plasmid content in Fig. 3 and Supplementary Fig. 6, we ran UMAP v0.5.1 (ref. ^86^) with the parameters ‘n_neighbors = 30, n_components = 2, min_dist = 0.15, metric = ‘jaccard’, random_state = 1’. The heat maps in Fig. 3 were generated using the heatmap.2 package in R, with agglomerative clustering using median linkage on Euclidean distances.

Third, to study the specific plasmids from PS974 and PS1110 in Fig. 5f and Supplementary Fig. 14 (contig names in Supplementary Table 11), we ran Bowtie 2 on each sequence separately. This set-up allowed every read to potentially align to multiple sequences, resulting in a more complete estimation of which metagenomes contained a plasmid. For the backbone sequences of these systems, we designated them as present in a metagenome if their detection was ≥0.95. For compound plasmids in PS1110 that encoded a chloramphenicol-resistance gene, we designated them as present in a metagenome if they satisfied an additional criterion that ≥0.95 of the resistance gene was covered by at least one read.

### Estimation of potential hosts for plasmids in metagenomes

We used two different formulae to calculate the ecological similarity between a plasmid and potential host: (1) the Pearson correlation in the abundance levels of the plasmid and host across metagenomes, and (2) a Jaccard index to represent the fraction of metagenomes that contain both the plasmid and host.

We estimated taxonomic abundances in every metagenome by running Kraken 2 (ref. ^87^) v2.1.2 with its standard database (https://github.com/DerrickWood/kraken2) and then refined the abundances using Bracken^88^ v2.5 (https://github.com/jenniferlu717/Bracken) with database parameters ‘-k 35 -l 96’. We ran Bracken (parameter ‘-r 96’) a separate time for every taxonomic rank: S1 (subspecies/strain), S (species), G (genus), F (family), O (order), C (class), P (phylum) and D (domain). The output of this analysis is a count of how many reads originated from each taxon.

To compare the metagenomic presence/absence of plasmids versus taxa, we calculated *M*_*P*_, the set of metagenomes where a plasmid *P* is detected at ≥95% (based on recruitment to the 68,350 non-redundant plasmids) and ${M}_{T}^{r}$, the set of metagenomes in which at least *r* reads originated from taxon *T* (based on Kraken and Bracken). For each plasmid, we attempted to find the best explanation of its ecological distribution by comparing the plasmid to every taxon using the Jaccard index and by scanning many possible read thresholds. More exactly, we used the following formula to represent the best possible explanation of the ecological distribution of a plasmid:

$$\mathop{\max }\limits_{T}\mathop{\max }\limits_{r}\,\mathrm{Jaccard}(P,\,T;\,r)$$

where

$$\mathrm{Jaccard}(P,\,T;\,r)=\frac{|{M}_{P}\cap {M\;}_{T}^{r}|}{|{M}_{P}\cup {M\;}_{T}^{r}|}$$

We evaluated 29 values for the threshold *r*, ranging from one to ten million reads in multiplicative increments of 10^1/4^. We ignored plasmids that were present in fewer than five metagenomes (that is, |*M*_*P*_| < 5) because it was probable that these plasmids would have a high Jaccard similarity to some taxon by random chance. For instance, we observed that many pairs of plasmids and taxa occur in exactly one and the same metagenome and thus they have a Jaccard index of one.

To compare continuous-valued abundances, we defined the abundance of a plasmid in a metagenome as the sum of coverage values across all sequence positions divided by sequence length, and we defined the abundance of a taxon as Bracken’s estimate of the number of reads originating from the taxon. If a plasmid had a detection of <95%, then we set its abundance to zero. If a taxon had <1,000 reads, then we set its abundance to zero. We ignored plasmids and taxa that had non-zero abundances in fewer than five metagenomes. For every pair of plasmid and taxon, we estimated the Pearson correlation between their abundance levels across metagenomes using FastSpar^89^ v1.0.0 (https://github.com/scwatts/fastspar), which is an improved implementation of the SparCC^90^. This method accounts for the compositional nature of the data—in which abundances reflect relative instead of absolute quantities—by assuming that the amount of correlations in a dataset is sparse. We ran FastSpar on the non-redundant set of predicted plasmids and ran it separately on the non-redundant set of reference plasmids.

We performed a more careful analysis of plasmid pDOJH10S and its cognate host *B. longum* DJO10A by performing a read recruitment of metagenomes to both sequences together, using Bowtie 2 and allowing every read to align at most once (Supplementary Fig. 7f and Supplementary Table 13). Following Utter et al.^91^, we applied a detection threshold of >50% to identify the presence of the plasmid or host: 365 metagenomes contained the plasmid, 818 metagenomes contained the cognate host and 336 metagenomes contained both genomes (Jaccard index = 0.40).

#### Keyword analysis of COGs and Pfams for plasmid functions

We labelled COGs and Pfams as plasmid-associated functions (Fig. 1e) if their database description contained any of following keywords as a substring: plasmid, toxin, replicat, integrase, transpos, recombinase, resolvase, relaxase, recombination, partitioning, mobilis, mobiliz, type IV, conjugal, conjugat, segregat, MobA, ParA, ParB and BcsQ. We labelled backbone and cargo genes as related to plasmid replication, transfer or maintenance if they were annotated to any plasmid-associated COG or Pfam (see the ‘Classification of cargo and backbone genes’ section).

To determine whether a predicted plasmid was ‘keyword-recognizable’ (Fig. 2c), we searched the plasmids for COGs and Pfams using a more restricted set of keywords (just plasmid and conjugation) instead of the keywords above.

### MobMess algorithm to de-replicate plasmids, remove assembly fragments and discover plasmid systems

The MobMess algorithm performs three tasks. It de-replicates plasmids that are nearly redundant to each other, it removes plasmids that seem to be assembly fragments and finally, it organizes plasmids together into evolutionary groups called plasmid systems. MobMess consists of several steps, as described below.

MobMess first performs an all-versus-all pairwise alignment of sequences using the MUMmer alignment package (v4.0.0rc1)^92^. All sequences are placed into a single fasta file and then aligned with ‘nucmer’ (parameters ‘--maxmatch --minmatch = 16’) to calculate local alignment blocks. Alignments are specified asymmetrically such that one sequence is designated as the query *q* and the other is the reference *r*. For every *q* and *r*, the alignment blocks calculated by nucmer are written to a separate file and then a subset of blocks is identified using ‘delta-filter’ (parameters ‘-q -r’) to create a one-to-one alignment.

Next, MobMess constructs a directed graph *G* where vertices are sequences and edges represent the containment of one sequence within another (Supplementary Fig. 8a). Formally, consider a query *q* and reference *r*. Let |*q*| be the length of *q*. For the *i*th alignment block between *q* and *r*, let *s*^*i*^, *e*^*i*^ and *δ*^*i*^ be the start position in *q*, end position in *q*, and number of alignment mismatches and indels, respectively. The following values summarize the information across all alignment blocks between *q* and *r*.

$$\begin{array}{cc}{\rm{Sum}}\,{\rm{of}}\,{\rm{block}}\,{\rm{lengths}} & L=\mathop{\sum}\limits_{i}{e}^{i}-{s}^{i}\end{array}$$

$$\begin{array}{cc}{\rm{Number}}\,{\rm{of}}\,{\rm{mismatches}}\,{\rm{and}}\,{\rm{indels}} & E=\mathop{\sum}\limits_{i}{\delta }^{i}\end{array}$$

$${\rm{Proportion}}\,{\rm{of}}\,{\rm{query}}\,{\rm{positions}}\,{\rm{covered}}\, C=\mathop{\sum }\limits_{j=1}^{|q|}\left\{\begin{array}{cc}1/|q| & {\rm{if}}\,\exists i\,{\rm{such}}\,{\rm{that}}\,{{s}}^{{i}}\le j\le {e}^{i}\\ 0 & {\rm{otherwise}}\end{array}\right.$$

$$\begin{array}{cc}{\rm{Local}}\,{\rm{sequence}}\,{\rm{identity}} & {I}_{\mathrm{local}}=(L-E\;)/L\end{array}$$

$$\begin{array}{cc}{\rm{Global}}\,{\rm{sequence}}\,{\rm{identity}} & {I}_{\mathrm{global}}=\end{array}{I}_{\mathrm{local}}\times C$$

MobMess creates a directed edge (*q*,*r*) in *G* if *I*_local_ and *C* are above user-specified thresholds. In this study we applied thresholds of *I*_local_ ≥ 0.9 and *C* ≥ 0.9 (Supplementary Information). In Supplementary Fig. 9, we re-ran MobMess using various thresholds on *I*_local_ and *C* (the same threshold was applied to *I*_local_ and *C* at the same time).

MobMess clusters sequences according to strongly connected components in *G*, calculated using igraph v0.8.2 (ref. ^93^) in Python. That is, two sequences *x* and *y* are placed in the same cluster if there exists a directed path from *x* to *y* and another from *y* to *x* in *G*. Intuitively, a cluster represents a set of sequences that are nearly identical to each other across nearly their entire lengths. MobMess then reduces *G* to another graph *H*, called the condensation graph, by contracting every cluster of sequences into a single vertex. A directed edge (*u*,*v*) exists in *H* if and only if there are sequences $x\in u$ and $y\in v$ where edge (*x,y*) exists in *G*. Note that *H* does not have any cycles. As proof by contradiction, if there were a cycle of clusters, then those clusters would have been in the same strongly connected component in *G* and hence, would have been merged into a single, larger cluster.

MobMess labels every cluster in *H* as one of the three following types: (1) a ‘backbone cluster’ if it has an outgoing edge and at least one of its member sequences is circular, (2) a ‘fragment cluster’ if it has an outgoing edge but none of its member sequences are circular or (3) a ‘maximal cluster’ if it does not have any outgoing edges. Intuitively, a maximal cluster represents the longest version of a plasmid observed in the data. In contrast, a backbone or fragment cluster represents a set of plasmids that are subsequences of other plasmids in a maximal cluster. The only difference between backbone and fragment clusters is that backbone clusters contain at least one circular plasmid (implying complete assembly), whereas fragment clusters do not contain any circular plasmids (suggesting that they are assembly fragments of the maximal cluster).

To de-replicate sequences, MobMess discards all fragment clusters and then chooses a representative sequence from every maximal and backbone cluster. A cluster’s representative is the sequence with the highest global sequence identity (*I*_global_) averaged across the set of alignments where that sequence is the reference and other sequences in the same cluster are the queries.

MobMess defines a plasmid system as a specific backbone cluster together with its ‘compound’ clusters, which are the set of non-fragment clusters connected to the backbone in *H*. Thus, there is a one-to-one correspondence between backbone clusters and plasmid systems. Note that systems can be nested within each other, because backbone clusters can be connected to each other in *H*. Thus, a backbone cluster can be the backbone that forms a given plasmid system and at the same time, it can also be a compound cluster with respect to an even smaller backbone that forms a different system. As another note, a maximal cluster can be a compound cluster of a system but it is also possible that some maximal clusters are not found in any system because they are not connected to any backbone clusters in *H*.

We ran MobMess to analyse the 226,194 predicted plasmid contigs. MobMess grouped the contigs into a total of 132,616 clusters. Of these, 64,266 clusters were ‘fragment clusters’ that contained 125,475 contigs, which we interpreted as assembly fragments of other predicted plasmids. We discarded these fragments from further analysis. The other 68,350 clusters were non-fragment clusters (that is, 1,169 backbone and 67,181 maximal clusters) and contained 100,719 contigs. Finally, MobMess identified 1,169 plasmid systems, which together represent 1,169 backbone and 63,926 maximal clusters (3,255 maximal clusters were excluded). See Supplementary Fig. 3 for a diagram of these numbers.

We ran MobMess separately on the 16,827 reference plasmid sequences, yielding 11,121 clusters. We assumed that all reference plasmids were circular and thus, there were no fragment clusters. We visualized networks with Cytoscape^94^ v3.8 and laid nodes out using the prefuse directed force layout^95^. Although we have focused on plasmids, MobMess could be applied to de-replicate and organize other mobile genetic elements into systems.

### Classification of cargo and backbone genes

We classified all genes on the backbone plasmids of a plasmid system as backbone genes. For genes on compound plasmids, we tested whether the genes shared any de novo family annotations with the genes on the backbone plasmids. If so, we classified those genes as backbone genes, otherwise as cargo genes. For this analysis, we used the 1,090,132 de novo families that we constructed from reference plasmids and chromosomes to train PlasX; we also used an additional set of 439,584 de novo families that we constructed by running the command MMseqs2 (--min-seq-id 0.05) on the genes from all plasmid sequences in this study (16,827 reference and 226,194 predicted plasmids). These additional families allowed us to capture gene families that might be absent in reference sequences but are conserved in predicted plasmids. Note that the classification of genes as backbone or cargo depends on which plasmid system is being considered. It is possible for a gene to be classified as a backbone gene with respect to one plasmid system and, at the same time, as a cargo gene with respect to another system. This is because a plasmid can be a backbone plasmid of a system and also a compound plasmid of a different system.

For every non-redundant compound plasmid in the system, we calculated the fraction of genes in the plasmid that were cargo genes. We then averaged this fraction across all non-redundant compound plasmids in the system to define the ‘cargo gene percentage’ of the system (Supplementary Fig. 12). Because every gene is either backbone or cargo, the percentage of backbone genes is 100% minus the cargo gene percentage.

For Fig. 4b and to analyse the content of backbone/cargo genes, we used a non-redundant and unambiguous set of 8,995 backbone and 24,168 cargo genes. To derive these sets of genes, we first considered the 47,172 genes encoded on the 4,424 non-redundant plasmids that were part of at least one plasmid system. Of these 47,172 genes, we used the 8,995 genes that were classified as backbone genes because they were encoded on a backbone plasmid and that were never classified as cargo genes in any plasmid system. Of these genes, 24.1% (2,169/8,995) had a plasmid-associated keyword in their COG/Pfam annotations (see the ‘Keyword analysis of COGs and Pfams for plasmid functions’ section). We also used the 24,168 genes that were always classified as cargo genes and never backbone genes in any plasmid system. Among these genes, 13.4% (3,229/24,168) had a plasmid-associated keyword. We excluded the 1,917 genes that were sometimes classified as backbone genes and other times cargo genes, depending on the system, from the analysis. We also excluded 12,092 genes that were on compound plasmids but were classified as backbone genes, as these genes are redundant with the backbone genes that were encoded on the backbone plasmid.

### Identification of antibiotic-resistance genes

We annotated antibiotic-resistance genes using two databases. First, we searched against a database of resistance protein family HMMs from Resfams^96^ (v1.2; dated 27 January 2015, ‘Core’ database at http://www.dantaslab.org/resfams). We used ‘anvi-run-hmms‘ from anvi’o^71^ to automate running ‘hmmsearch’ from HMMER^75^ 3.3.2 and apply an *e*-value cutoff of 1 × 10^−10^. Second, we ran rgi (v5.2.0; https://github.com/arpcard/rgi) to search for similarity in the CARD database of resistance genes^97^. We removed CARD hits that were labelled as ‘Loose’ and kept those labelled as ‘Perfect’ or ‘Strict’. We removed any Resfams or CARD hits that contained the keywords ‘transcription’, ‘regulat’ or ‘modulat’ in their database description to avoid cases (for example, TetR protein) where the hit is a gene that regulates the expression of another resistance gene but does not perform the molecular process that confers resistance. We categorized hits into major antibiotic-resistance classes by searching for the following keywords in their functional descriptions: lincosamide, macrolide, erythromycin, chloramphenicol, aminoglycoside, streptothricin, glycopeptide, efflux pump, beta-lactamase, nitroimidazole, tetraycyline, quinolone and sulfonamide. In addition, we searched the extra keywords ‘Van’ and ‘VanZ’ to identify glycopeptide resistance; ‘efflux’, ‘permease’ and ‘pump’ to identify efflux pumps; and ‘TetX’ to identify tetracycline resistance.

### High-molecular-weight DNA extraction, long-read sequencing and determination of circularity through long reads

We employed a long-read sequencing strategy on two *B. fragilis* cultivars from two patients (p-214 and n-216, previously described by Vineis and colleagues^43^). We extracted total genomic high-molecular-weight DNA using one of two methods. For *B. fragilis* p-214, we used the Qiagen Genomic Tip 20/G procedure (also known as Method #4/GT) as previously described^98^ on a 10 ml overnight BHIS broth culture. For *B. fragilis* n-216, we used a phenol–chloroform protocol on 25 ml overnight BHIS broth cultures. Libraries were prepared using a Rapid barcoding kit (SQK-RBK004) and the standard protocols from Oxford Nanopore Technologies, with a few modifications. For *B. fragilis* p-214, DNA fragmentation was performed on 6 µg DNA (in 30 µl) using five passes through a 22 G needle. A total of 1.5 µg (in 7.5 µl) genomic DNA (Supplementary Table 12), based on sample availability, plus 2.5 µl fragmentation mix was used as input. We sequenced the samples for 72 h using a single R9.4/FLO-MIN106 flow cell (Oxford Nanopore Technologies). For *B. fragilis* n-216, DNA fragmentation was performed on 10 µg DNA (in 250 µl) using ten passes through a 22 G needle. A total of input 0.32–0.44 µg genomic DNA (in 8.5 µl) plus 1.5 µl fragmentation was used as input. The samples were sequenced for 72 h using a single R9.4/FLO-MIN106 flow cell. We used Guppy (v4.0.15) for all post-run base calling, sample de-multiplexing and the conversion of raw FAST5 files to FASTQ files.

To determine circularity, we used BLAST to align the long reads with a minimum quality score of seven to our predicted plasmid sequences following a previously described approach^35^. During assembly, all DNA short reads are assembled as linear sequences even if they are circular elements. Circular elements have an artificial breakpoint to represent them as linear sequences, and this breakpoint can happen anywhere on the sequence depending on the assembly method. We identified and manually confirmed 500 long reads that aligned completely to a plasmid but not to the host chromosome (Supplementary Figs. 4a and 5b). We tested for the presence of an artificially introduced breakpoint by visualizing these alignments on the sequence as if it were assumed to be a circular element (Fig. 5a). If the sequence was indeed circular, the long reads would overlap each other and ‘wrap around’ the entire circumference of the sequence. In other words, all nucleotide positions of the sequence would be covered by at least one read and there would also exist a read that spans the breakpoint by aligning to both sides of the breakpoint. This property ensures the breakpoint is artificial and hence, the sequence is a circular element. Inversely, this property does not hold when the breakpoint is not artificial (that is, the sequence is actually an assembly fragment or linear element). Some of these long reads aligned across the artificial contig breakpoint, indicating that these plasmids were extrachromosomal and circular.

### Transfer of predicted plasmid between microbial populations

We streaked *B. fragilis* p-214 (donor, carries erythromycin resistance on pFIJ0137_1; one of 14 isolates from Vineis et al.^43^) and *B. fragilis* 638R (recipient, rifampicin-resistant) in duplicate onto plates with brain-heart infusion agar supplemented with hemin and vitamin K (BHIS). We picked colonies and incubated them anaerobically in 5 ml BHIS medium at 37 °C for 20 h. Although pFIJ0137_1 lacks conjugation machinery, it contains two relaxases (blue genes in Supplementary Fig. 4) and thus could be mobilized by different conjugative apparatus in the host cell. To mate the donor to the recipient, 250 μl of donor cells were pelleted in a centrifuge at 5,000*g*. We discarded the supernatant and resuspended the donor in 1 ml of the recipient culture. The cells were again pelleted at 5,000*g* and then resuspended in 25 μl BHIS medium. The cells were spotted onto BHIS agar plates and incubated anaerobically for 24 h. The cells were then resuspended in 1 ml BHIS; 250 μl of this suspension was plated onto BHIS plates containing 8 μg ml^−1^ rifampicin and 25 μg ml^−1^ erythromycin to select for *B. fragilis* 638R recipients of pFIJ0137_1. Duplicate plates had approximately 300 colonies each. Plating the donor or recipient alone on rifampicin–erythromycin plates resulted in no colonies, thereby confirming that the transformants were not spontaneous mutants to either antibiotic. Two transformant colonies were re-streaked onto fresh BHIS plates containing 8 μg ml^−1^ rifampicin and 25 μg ml^−1^ erythromycin. Through short-read sequencing of the donor, recipient and resulting transconjugants, and by employing a read-recruitment analysis, we confirmed that pFIJ0137_1 transferred from *B. fragilis* p-214 to *B. fragilis* 638R (Supplementary Fig. 4).

### Short-read sequencing of isolate genomes and confirmation of plasmid transfer

We cultured *B. fragilis* p-214 donor, naive *B. fragilis* 638R and *B. fragilis* 638R transconjugants containing pFIJ0137_1 for 20 h. Libraries of these strains were prepared using 100 ng genomic DNA and a QIAseq FX DNA library kit (Qiagen). The DNA was fragmented enzymatically into smaller fragments and the desired insert size was achieved by adjusting the fragmentation conditions. The fragmented DNA was end repaired and ‘A’s were added to the 3′ ends to stage inserts for ligation. During the ligation step, Illumina-compatible Unique Dual Index adaptors were added to the inserts and the prepared library was PCR amplified. The amplified libraries were purified, and quality control was performed using a TapeStation. The libraries were sequenced on an Illumina MiSeq platform using a v2 cassette to generate 2 × 250 bp reads. To confirm the transfer of pFIJ0137_1, we individually recruited reads from the *B. fragilis* p-214 donor, naive *B. fragilis* 638R and *B. fragilis* 638R transconjugants to the pFIJ0137_1 reference sequence. We used anvi’o to create contigs and profile databases (as described earlier) and visualized these results with the command ‘anvi-interactive’. We independently confirmed the presence of pFIJ0137_1 by assembling genomes using SPAdes^99^ with default parameters.

### Reporting summary

Further information on research design is available in the Nature Portfolio Reporting Summary linked to this article.

### Supplementary information

Supplementary Information Supplementary Figs. 1–14, Supplementary Notes and Supplementary References.

Reporting Summary

Supplementary Table 8 Summary of predicted plasmids (model scores, orthogonal support, circularity and NCBI blast results).

Supplementary Tables 1–7 and 9–13

## Data Availability

Reproducible analyses of reference plasmids and chromosomes are available at 10.5281/zenodo.5732024. The PlasX model as well as our analyses of known and predicted plasmids are available at 10.5281/zenodo.5843600. For all metagenomes, we have compiled the contigs, taxonomic abundances and PlasX scores at 10.5281/zenodo.8175278, gene calls at 10.5281/zenodo.5730987 and gene annotations at 10.5281/zenodo.5731658. We have deposited long and short sequencing reads from *B. fragilis* isolates into the NCBI Sequence Read Archive (PRJNA782184). We obtained a list of 16,168 plasmids from the 2019_03_05 version of PLSDB^68^. We also downloaded the entire collection of 13,471 complete bacterial genome assemblies from NCBI RefSeq (accessed 26 October 2019), using instructions at https://www.ncbi.nlm.nih.gov/genome/doc/ftpfaq/#allcomplete (ref. ^69^). We also downloaded the more recent 2021_06_23_v2 version of PLSDB, which contains 34,513 plasmid sequences. We downloaded the collection of all ICE sequences (*n* = 552) from ICEberg^36^ 2.0 (https://db-mml.sjtu.edu.cn/ICEberg/; accessed 30 September 2022). We also downloaded 455 prophage sequences from the NCBI Virus data portal (https://www.ncbi.nlm.nih.gov/labs/virus; accessed 30 September 2022). We downloaded fastq files for 1,782 short-read and paired-end metagenomes from the NCBI Sequence Read Archive using the program ‘fastq-dump’. The metagenomes and original studies are listed in Supplementary Table 7. We annotated antibiotic-resistance genes using two databases. First, we searched against a database of resistance protein family HMMs from Resfams^96^ (v1.2, dated 27 January 2015; ‘Core’ database at http://www.dantaslab.org/resfams). Second, we ran rgi (v5.2.0; https://github.com/arpcard/rgi) to search for similarity in the CARD database of resistance genes.

## Code availability

We have released two open-source software packages, PlasX^100^ and MobMess^101^, along with detailed installation and usage instructions. We used the program anvi-run-workflow with --workflow contigs implemented^70^ in anvi’o^71^ v7.1, which uses Snakemake^72^ to execute previously defined steps (https://merenlab.org/anvio-workflows/) and to generate anvi’o contigs-db files (https://anvio.org/m/contigs-db). These steps include first running Prodigal^73^ to call genes and then running DIAMOND^74^ v2.0 and HMMER^75^ v3.3 on amino acid sequences to determine gene functions against the Clusters of Orthologous Groups (COGs)^30^ and Protein Family Database^31^ models (Pfams) v32.0, respectively. We clustered genes using MMSeqs2 (ref. ^76^; v10.6d92c); identified sequence subtypes using mash^79^ (v2.2.2); analysed plasmids using PlasClass^20^ (v0.1.0-2-gb80a4f4), PPR-Meta^32^, Platon^33^, Deeplasmid^27^ (Docker image sha256:10809927e2c8a14cf86231801b804b0bd4bddf600821d17fd8b7e41a15c562c0) and MOB-suite^25^ (v3.0.1); visualized networks using Cytoscape^94^ (v3.8); performed taxonomic assignment using Kraken 2 (ref. ^87^; v2.1.2) and Bracken^88^ (v2.5); and ran correlation analysis using FastSpar^89^ (v1.0.0).
