# Supplementary material for: Diverse plasmid systems and their ecology across human gut metagenomes revealed by PlasX and MobMess
Source: Nat Microbiol. 2024 Mar 4;9(3):830–47. doi: 10.1038/s41564-024-01610-3 (PMC10914615; doi:10.1038/s41564-024-01610-3)
Supplement: Supplementary file 2 — Reporting Summary [file 41564_2024_1610_MOESM2_ESM.pdf]

nature portfolio

Corresponding author(s): Michael K. Yu; A. Murat Eren

Last updated by author(s): Nov 8, 2023

Reporting Summary

Nature Portfolio wishes to improve the reproducibility of the work that we publish. This form provides structure for consistency and transparency in reporting. For further information on Nature Portfolio policies, see our Editorial Policies and the Editorial Policy Checklist.

Statistics

For all statistical analyses, confirm that the following items are present in the figure legend, table legend, main text, or Methods section.

n/a	Confirmed	
☐	☒	The exact sample size (*n*) for each experimental group/condition, given as a discrete number and unit of measurement
☐	☒	A statement on whether measurements were taken from distinct samples or whether the same sample was measured repeatedly
☒	☐	The statistical test(s) used AND whether they are one- or two-sided<br>*Only common tests should be described solely by name; describe more complex techniques in the Methods section.*
☒	☐	A description of all covariates tested
☒	☐	A description of any assumptions or corrections, such as tests of normality and adjustment for multiple comparisons
☐	☒	A full description of the statistical parameters including central tendency (e.g. means) or other basic estimates (e.g. regression coefficient) AND variation (e.g. standard deviation) or associated estimates of uncertainty (e.g. confidence intervals)
☐	☒	For null hypothesis testing, the test statistic (e.g. *F*, *t*, *r*) with confidence intervals, effect sizes, degrees of freedom and *P* value noted<br>*Give P values as exact values whenever suitable.*
☒	☐	For Bayesian analysis, information on the choice of priors and Markov chain Monte Carlo settings
☒	☐	For hierarchical and complex designs, identification of the appropriate level for tests and full reporting of outcomes
☐	☒	Estimates of effect sizes (e.g. Cohen's *d*, Pearson's *r*), indicating how they were calculated

Our web collection on statistics for biologists contains articles on many of the points above.

Software and code

Policy information about availability of computer code

Data collection

All details about data and code used are described in the Methods section and Supplementary Information.

We have released two open-source software packages, PlasX (https://github.com/michaelkyu/plasx) and MobMess (https://github.com/michaelkyu/mobmess), along with detailed installation and usage instructions.

Data analysis

All details about data and code used are described in the Methods section and Supplementary Information.

We have released two open-source software packages, PlasX (https://github.com/michaelkyu/plasx) and MobMess (https://github.com/michaelkyu/mobmess), along with detailed installation and usage instructions.

We used the program `anvi-run-workflow` with `--workflow contigs` implemented in anvi'o v7.1, which uses Snakemake to execute previously defined steps (https://merenlab.org/anvio-workflows/) and to generate anvi'o contigs-db files (https://anvio.org/m/contigs-db). These steps include first running Prodigal to call genes and then running DIAMOND v2.0 and HMMER v3.3 on amino acid sequences to determine gene functions against the Cluster of Orthologous Groups of proteins (COGs) and Protein Family Database models (Pfams) v32.0, respectively.

We clustered genes using MMSeqs2 (v10.6d92c); identified sequence subtypes using mash (v2.2.2); analyzed plasmids using PlasClass v0.1.0-2-gb80a4f4, PPR-Meta, Platon, Deeplasmid (Docker image sha256:10809927e2c8a14cf86231801b804b0bd4bddf600821d17fd8b7e41a15c562c0), and MOB-suite v3.0.1); visualized networks using Cytoscape v3.8, performed taxonomic assignment using kraken2 v2.1.2 and bracken v2.5, and ran correlation analysis using FastSpar v1.0.0.

For manuscripts utilizing custom algorithms or software that are central to the research but not yet described in published literature, software must be made available to editors and reviewers. We strongly encourage code deposition in a community repository (e.g. GitHub). See the Nature Portfolio guidelines for submitting code & software for further information.

Data

Policy information about availability of data

All manuscripts must include a data availability statement. This statement should provide the following information, where applicable:

- Accession codes, unique identifiers, or web links for publicly available datasets
- A description of any restrictions on data availability
- For clinical datasets or third party data, please ensure that the statement adheres to our policy

All details about data and code used are described in the Methods section and Supplementary Information.

Reproducible analyses of reference plasmids and chromosomes are available at doi:10.5281/zenodo.5732024. The PlasX model as well as our analyses of known and predicted plasmids are available at doi:10.5281/zenodo.5843600. For all metagenomes, we have compiled the contigs, taxonomic abundances, and PlasX scores at doi:10.5281/zenodo.8175278, gene calls at doi:10.5281/zenodo.5730987, and gene annotations at doi:10.5281/zenodo.5731658. We have deposited long and short sequencing reads from B. fragilis isolates into the NCBI Sequence Read Archive (PRJNA782184).

We obtained a list of 16,168 plasmids from the 2019_03_05 version of PLSDB. We also downloaded the entire collection of 13,471 complete bacterial genome assemblies from NCBI RefSeq on October 26, 2019, using instructions at https://www.ncbi.nlm.nih.gov/genome/doc/ftpfaq/#allcomplete. We also downloaded the more recent 2021_06_23_v2 version of PLSDB, which contains 34,513 plasmid sequences. We downloaded the collection of all ICE sequences (n=552) from ICEberg 2.0 at https://db-mml.sjtu.edu.cn/ICEberg/ on September 30, 2022. We also downloaded 455 prophage sequences from the NCBI Virus data portal (https://www.ncbi.nlm.nih.gov/labs/virus) on September 30, 2022.

We downloaded fastq files for 1,782 short-read and paired-end metagenomes from the National Center for Biotechnology Information (NCBI) Sequence Read Archive (SRA) using the program `fastq-dump`. The metagenomes and original studies are listed in Table S7.

We annotated antibiotic resistance genes using two databases. First, we searched against a database of resistance protein family HMMs from Resfams (v1.2, dated 2015-01-27, 'Core' database at http://www.dantaslab.org/resfams). Second, we ran rgi (v5.2.0, https://github.com/arpcard/rgi) to search for similarity in the CARD database of resistance genes.

Human research participants

Policy information about studies involving human research participants and Sex and Gender in Research.

Reporting on sex and gender	N/A
Population characteristics	N/A
Recruitment	N/A
Ethics oversight	N/A

Note that full information on the approval of the study protocol must also be provided in the manuscript.

Field-specific reporting

Please select the one below that is the best fit for your research. If you are not sure, read the appropriate sections before making your selection.

☒ Life sciences ☐ Behavioural & social sciences ☐ Ecological, evolutionary & environmental sciences

For a reference copy of the document with all sections, see nature.com/documents/nr-reporting-summary-flat.pdf

Life sciences study design

All studies must disclose on these points even when the disclosure is negative.

Sample size	One plasmid was tested for transfer. Two plasmids were sequenced with long-read sequencing. No sample size calculations were performed, as these experiments served as anecdotes to confirm model predictions.
Data exclusions	No data was excluded.
Replication	Bacterial plasmid transfer experiments were conducted in duplicates. Both duplicates were successful.
Randomization	Not applicable because of the small sample size.
Blinding	Not applicable because no blinding was needed to confirm positive results.

Reporting for specific materials, systems and methods

We require information from authors about some types of materials, experimental systems and methods used in many studies. Here, indicate whether each material, system or method listed is relevant to your study. If you are not sure if a list item applies to your research, read the appropriate section before selecting a response.

Materials & experimental systems

n/a	Involved in the study
☒	☐ Antibodies
☒	☐ Eukaryotic cell lines
☒	☐ Palaeontology and archaeology
☒	☐ Animals and other organisms
☒	☐ Clinical data
☒	☐ Dual use research of concern

Methods

n/a	Involved in the study
☒	☐ ChIP-seq
☒	☐ Flow cytometry
☒	☐ MRI-based neuroimaging
